# Efficacy of the early start Denver model combined with the TEACCH program in children with autism spectrum disorder

**DOI:** 10.3389/fpsyt.2025.1669476

**Published:** 2025-11-18

**Authors:** Ling-Ling Ma, Qian-Qian Lv, Yao Xiao

**Affiliations:** Department of Rehabilitation, Hangzhou Children’s Hospital, Hangzhou, Zhejiang, China

**Keywords:** autism spectrum disorder, early start Denver model, TEACCH program, cognitive development, behavioral intervention

## Abstract

**Background:**

Early intervention plays a crucial role in improving outcomes for children with autism spectrum disorder (ASD). The Early Start Denver Model (ESDM) and the Treatment and Education of Autistic and Communication-Handicapped Children (TEACCH) program are commonly used approaches. This study aimed to evaluate whether combining ESDM with TEACCH provides greater clinical benefits than ESDM alone in young children with ASD.

**Methods:**

A retrospective observational study was conducted involving 264 children aged 24–60 months diagnosed with ASD. Participants were divided into two groups based on treatment period: the control group (n = 128) received ESDM-only therapy, while the observation group (n = 136) received ESDM combined with TEACCH over six months. Outcomes were assessed using the Autism Treatment Evaluation Checklist (ATEC) and the Psycho-Educational Profile, Third Edition (PEP-3). Baseline comparability was confirmed by the Chinese version of the Childhood Autism Rating Scale (CCARS).

**Results:**

Both groups showed significant within-group improvement after intervention, but the combined observation group demonstrated greater gains. Post-treatment ATEC scores decreased from 84.56 ± 20.90 to 68.76 ± 17.96 versus 92.84 ± 18.20 to 84.91 ± 17.50 in controls (between-group difference = 16.32 ± 4.35; P < 0.001; Hedges’ g = 0.45). Cognitive scores on the PEP-3 improved by 11.31 points in the observation group compared to 8.15 in controls (P = 0.026). Reductions in maladaptive behaviors also favored the combined intervention (P = 0.036).

**Conclusions:**

The integrated ESDM and TEACCH intervention was more effective than ESDM alone in enhancing cognitive development and reducing symptom severity in young children with ASD.

## 1 Introduction

Autism Spectrum Disorder (ASD) is a complex neurodevelopmental condition characterized by persistent deficits in social communication and interaction, alongside restricted, repetitive patterns of behavior, interests, or activities. The global prevalence of ASD has increased significantly over recent decades, currently estimated at approximately 1 in 100 children, according to the World Health Organization (1). The clinical heterogeneity of ASD presents substantial challenges in early diagnosis and individualized intervention, underscoring the necessity for evidence-based, multifaceted therapeutic approaches tailored to the developmental needs of affected children (2, 3).

Early intervention is widely recognized as a cornerstone in optimizing developmental outcomes in children with ASD (4). Among various early intervention models, the Early Start Denver Model (ESDM) and the Treatment and Education of Autistic and Communication-Handicapped Children (TEACCH) approach have both demonstrated effectiveness across multiple domains (5). ESDM is a comprehensive, play-based, and relationship-focused early behavioral intervention designed for children aged 12 to 48 months. Rooted in principles of applied behavior analysis (ABA), ESDM integrates developmental and behavioral techniques to promote social communication, cognitive, and language skills in naturalistic settings (6, 7). In contrast, the TEACCH program emphasizes structured teaching and environmental modifications to facilitate autonomy, predictability, and task engagement in individuals with ASD. TEACCH is particularly effective in enhancing adaptive behavior and reducing behavioral rigidity through visual supports, individualized routines, and structured learning environments. Although both the Early Start Denver Model (ESDM) and the Treatment and Education of Autistic and Communication-Handicapped Children (TEACCH) program have demonstrated positive outcomes in children with autism spectrum disorder (ASD), existing studies have primarily evaluated these interventions in isolation (8–10). To date, limited research has investigated the clinical effectiveness of combining ESDM and TEACCH within a single, integrated treatment framework (11, 12). In particular, there is a lack of controlled studies directly comparing outcomes between ESDM alone and ESDM combined with TEACCH, which hinders our understanding of the potential added value of such a combined approach (13).

In this context, the present study aims to evaluate the clinical efficacy of a combined intervention strategy incorporating both the Early Start Denver Model and the TEACCH program in children diagnosed with ASD. By assessing the outcomes of this integrated approach, the current research seeks to contribute empirical evidence to inform early intervention strategies and refine multidisciplinary treatment protocols for children with ASD. The findings are expected to enhance our understanding of the potential synergistic effects of combining developmental and structured teaching paradigms in early therapeutic interventions for ASD.

## 2 Methods

### 2.1 Study design

This retrospective observational study was conducted to evaluate the clinical efficacy of the ESDM alone and in combination with the TEACCH program in children diagnosed with ASD. The study population comprised patients treated in the Department of Rehabilitation of our institution between January 2021 and December 2024. Inclusion criteria were as follows: (1) a diagnosis of ASD confirmed by qualified developmental pediatricians based on the Diagnostic and Statistical Manual of Mental Disorders, Fifth Edition (DSM-5) criteria; (2) age between 24 and 60 months at the time of enrollment; (3) no prior systematic behavioral intervention before receiving treatment at our institution; and (4) complete baseline and follow-up assessment data. Exclusion criteria included: (1) comorbid neurological disorders such as epilepsy or cerebral palsy; (2) significant sensory impairments (e.g., blindness or deafness) that would interfere with behavioral assessment or therapy delivery; (3) Cognitive ability was assessed at baseline using the Chinese revision of the Wechsler Intelligence Scale for Children administered by certified clinical psychologists (14). Children with IQ <40 were excluded; and (4) any major psychiatric disorder other than ASD. Beginning in January 2021, ESDM was formally implemented as the standard intervention. Children with ASD who received ESDM-based therapy between January 2021 and December 2022 (n = 128) were assigned to the control group. From January 2023 onward, the TEACCH program was incorporated into the existing ESDM framework, and children treated between January 2023 and December 2024 (n = 136) were classified into the observation group. Informed consent was obtained from all subjects and/or their legal guardian(s). The study was reviewed and approved by the hospital’s ethics committee. All procedures followed relevant guidelines and adhered to the ethical principles of the Declaration of Helsinki. Participant data were anonymized prior to analysis to ensure confidentiality and privacy.

### 2.2 ESDM-based intervention in the control group

Children in the control group received intervention based exclusively on the ESDM, a relationship-focused, developmentally informed, and behaviorally based approach designed for young children with ASD. A multidisciplinary intervention team was assembled for each child, consisting of trained therapists, healthcare professionals, and primary caregivers. Each child underwent a comprehensive developmental assessment encompassing cognitive abilities, expressive and receptive language, motor coordination, and social engagement. Based on these assessments, individualized intervention plans were developed with clearly defined rehabilitation objectives. Intervention strategies involved identifying and leveraging the child’s interests to promote engagement, providing timely and responsive feedback during communication, and emphasizing the development of expressive language during social interactions. Positive behavior support techniques were used to encourage the acquisition and generalization of new behaviors and skills. Family involvement was an integral part of the intervention process; caregivers received training to reinforce therapeutic goals in daily life. Specific skill domains targeted during the intervention included imitation, comprehension, verbal communication, social reciprocity, play behaviors, and self-care abilities. Each child received 2-hour daily sessions, six days per week, over a six-month period.

### 2.3 Combined ESDM and TEACCH intervention in the observation group

Children in the observation group received the same ESDM-based intervention as the control group, supplemented by the TEACCH program, which emphasizes structured teaching and environmental adaptation. The TEACCH strategies used in this study were grounded in its core principles of structured teaching, visual clarity, routine building, and individualized task design aimed at fostering autonomy and behavioral regulation in children with ASD. The observation group also received intervention six days per week for six consecutive months, allowing for direct comparison with the ESDM-only control group to evaluate the potential synergistic effects of the combined approach. The combined intervention targeted five core components:

1. Structured Physical Environment: Therapy spaces were functionally divided into specific zones, including areas for play, group instruction, individual learning, storage, and meals, to enhance spatial predictability and reduce environmental confusion.
2. Visual Structure: Clear visual cues such as colors, shapes, and pictorial aids were used to organize materials and guide learning tasks. Instructional visuals, including sequences for daily routines (e.g., handwashing, table manners, greeting peers), supported comprehension and behavior regulation.
3. Establishment of Daily Routines: Children were guided to develop consistent routines in both learning and daily living contexts. Social behaviors such as greeting others and expressing gratitude were practiced to promote social normalization.
4. Activity Schedules: Individualized daily schedules were created using photos, icons, or simple text, depending on each child’s developmental level. These schedules were posted in both the child’s activity area and the central program board to ensure consistent guidance throughout the day.
5. Individual Work Systems: Tailored task systems were developed based on each child’s functional level, incorporating visual and environmental structure, routine, and task predictability to promote autonomy and task completion.

### 2.4 Intervention fidelity

Both the ESDM and TEACCH interventions were implemented in our institution following standardized clinical procedures derived from publicly available guidelines and professional training resources. All therapists involved had received structured training in the core principles and techniques of both programs through institution-approved professional development activities. Training was conducted using publicly available resources, including ESDM guidance documents from early intervention platforms such as Foundations (UK), and was overseen by senior clinicians with extensive experience in developmental therapy.

Fidelity to intervention protocols was supported through multiple mechanisms, including standardized session planning templates, family coaching modules, and regular peer or supervisory review of clinical records. The same team of therapists trained in both ESDM and TEACCH strategies provided services to both study groups. As the study was based on retrospective review of routine clinical care, therapists were unaware of any subsequent research use of their sessions, minimizing potential bias in treatment delivery.

### 2.5 Data collection and assessment tools

Baseline demographic and clinical data were retrospectively extracted from the electronic medical records of all participants. Variables collected included age, sex, parental education, household income, family history of neurodevelopmental disorders, and relevant perinatal factors. Information on comorbid medical or psychiatric conditions (e.g., epilepsy, attention-deficit/hyperactivity disorder, language delay) was also recorded when available. Cognitive ability was assessed at baseline using the Chinese revision of the Wechsler Intelligence Scale for Children administered by certified clinical psychologists (14).

In addition to demographic and clinical characteristics, standardized, validated instruments widely used in autism spectrum disorder (ASD) research and clinical practice were employed to evaluate baseline status and treatment outcomes. All assessment tools were administered in their Chinese-translated and culturally adapted versions, with established reliability and validity for the Chinese pediatric population. Trained clinicians or research staff performed the assessments following standardized protocols, and all scoring was verified by senior developmental specialists to ensure consistency and objectivity.

1. Chinese version of Childhood Autism Rating Scale (CCARS) (15): Prior to intervention, all children underwent diagnostic evaluation using the CCARS, a clinician-administered instrument designed to differentiate children with ASD from those with other developmental disorders and to assess the severity of autism-related behaviors. The scale includes 15 items covering multiple symptom domains, including verbal and non-verbal communication, sensory responses, emotional regulation, and relationship to people. Each item is rated on a 4-point Likert scale (1 to 4), yielding a total score ranging from 15 to 60. According to standard cutoff thresholds, scores <30 indicate no ASD, 30–36 suggest mild-to-moderate ASD, and 37–60 reflect severe ASD.
2. Autism Treatment Evaluation Checklist (ATEC) (16): To evaluate treatment effectiveness, the ATEC was administered both before and after the intervention. Originally developed by Rimland and Edelson, the ATEC is a parent-reported scale designed to monitor changes in core ASD symptoms over time. The checklist consists of 77 items grouped into four subscales: Speech/Language/Communication (0–28 points), Sociability (0–40 points), Sensory/Cognitive Awareness (0–36 points), and Health/Physical/Behavior (0–75 points), for a total possible score of 0–179. Higher total and subscale scores reflect greater symptom severity. The scale is especially useful for longitudinal monitoring of therapeutic response.
3. Psycho-Educational Profile, Third Edition (PEP-3) (17): The PEP-3, was used to assess the developmental functioning and behavioral characteristics of children with ASD across multiple domains. Originally developed by Schopler and colleagues and revised in 2005, the PEP-3 is designed to identify uneven cognitive and behavioral profiles commonly seen in ASD. The PEP-3 includes three main components: (1) direct assessment of developmental and behavioral subtests, (2) caregiver report, and (3) composite scores. Domains assessed include communication, motor skills, maladaptive behavior, and social reciprocity. Lower scores are indicative of more pronounced developmental delays and more severe autistic symptoms.

### 2.6 Statistical analysis

All statistical analyses were performed using IBM SPSS Statistics for Windows, version 26.0 (IBM Corp., Armonk, NY, USA). Data were first examined for normality using the Kolmogorov–Smirnov test. Continuous variables were expressed as mean ± standard deviation (SD) if normally distributed, or as median with interquartile range (IQR) for non-normally distributed data. Categorical variables were summarized as frequencies and percentages. Between-group comparisons for continuous variables were conducted using independent samples t-tests or the Mann–Whitney U test, as appropriate. Categorical variables were analyzed using the chi-square test or Fisher’s exact test. Within-group comparisons before and after the intervention were performed using paired t-tests or Wilcoxon signed-rank tests, depending on the data distribution. Effect sizes (Cohen’s d) were calculated for primary and key secondary outcomes to aid clinical interpretation. Given the multiple comparisons across PEP-3 subdomains, the Benjamini–Hochberg false discovery rate (FDR) procedure was applied to control for Type I error inflation. To further account for potential baseline confounding, analysis of covariance (ANCOVA) models were performed, adjusting for baseline scores, age, and sex. In addition, propensity score weighting (inverse probability of treatment weighting, IPTW) was conducted using key demographic and clinical variables (age, sex, baseline CCARS, baseline ATEC, and baseline PEP-3 cognition scores) to perform sensitivity analyses. Both adjusted analyses and IPTW confirmed the robustness of the main results. All statistical tests were two-tailed, with a P-value < 0.05 considered statistically significant. For analysis with FDR correction, q-values < 0.05 were regarded as statistically significant.

## 3 Results

### 3.1 Baseline characteristics

A total of 264 children were included (128 in the control group and 136 in the observation group). The two groups were comparable at baseline with respect to sex distribution (P = 0.526), age (P = 0.129), and CCARS scores (P = 0.814). No significant differences were observed in baseline IQ, socioeconomic indicators (parental education, household income), or the prevalence of comorbid conditions, including epilepsy, ADHD symptoms, and language delay. Family history of ASD was rare and similarly distributed between groups. Overall, baseline demographic and clinical characteristics were balanced, supporting comparability prior to intervention (**Table 1**).

**Table 1:** Baseline characteristics of participants in the control and observation groups.

Variable	Control group (n = 128)	Observation group (n = 136)	Statistics (t/χ²/Z)	P-value
Gender (male/female), n	102/26	105/31	χ² = 0.402	0.526
Age (months), mean ± SD	56.02 ± 17.89	52.76 ± 16.85	t = 1.525	0.129
CCARS, median (IQR)	34.45 (32.00–43.25)	36.29 (32.15–45.22)	Z = 0.235	0.814
IQ, mean ± SD	72.41 ± 12.33	73.15 ± 11.98	t = –0.482	0.630
Parental education ≥ college, n (%)	39 (30.5%)	44 (32.4%)	χ² = 0.098	0.754
Household income ≥ median level, n (%)	41 (32.0%)	48 (35.3%)	χ² = 0.259	0.611
Comorbid conditions, n (%)	21 (16.4%)	19 (14.0%)	χ² = 0.252	0.616
– Epilepsy	6 (4.7%)	5 (3.7%)		
– ADHD symptoms	9 (7.0%)	8 (5.9%)		
– Language delay	12 (9.4%)	11 (8.1%)		
Family history of ASD, n (%)	5 (3.9%)	6 (4.4%)	χ² = 0.037	0.84

CCARS, Chinese version of Childhood Autism Rating Scale; IQ, Intelligence Quotient; IQR, Interquartile Range; SD, Standard Deviation; ASD, Autism Spectrum Disorder.

To further account for potential confounding, adjusted analyses were performed. Covariance models controlling for baseline values and demographic variables confirmed the absence of significant baseline differences. In addition, sensitivity analyses using propensity score weighting yielded consistent results, supporting the robustness of baseline comparability between groups (**Supplementary Table 1**).

### 3.2 Changes in ATEC scores

Analysis of within‐group changes in ATEC scores demonstrated statistically significant reductions following intervention in both cohorts. In the control group (n = 128), mean ATEC scores decreased from 92.84 ± 18.20 at baseline to 84.91 ± 17.50 after treatment (t = 3.553, P = 0.001). The observation group (n = 136) exhibited an even greater decline, with scores falling from 84.56 ± 20.90 pre-treatment to 68.76 ± 17.96 post-treatment (t = 6.687, P < 0.001). Between‐group comparisons revealed no significant difference at baseline (mean difference 8.28 ± 5.20; t = 1.865, P = 0.065), confirming comparable starting points. However, post-treatment ATEC scores were significantly lower in the observation group than in controls (mean difference 16.32 ± 4.35; t = 3.647, P < 0.001), indicating superior improvement in the combined ESDM+TEACCH intervention (**Table 2**). At post-treatment, the between-group effect size for ATEC total favored ESDM+TEACCH (Hedges’ g = 0.45, 95% CI 0.20–0.69) (**Supplementary Table 2**).

**Table 2:** Comparison of pre- and post-treatment ATEC scores within and between groups (mean ± SD).

Group/comparison	Time point	Score (mean ± SD)	t Value	P Value
Control Group (n = 128)	Pre-treatment	92.84 ± 18.20	—	—
	Post-treatment	84.91 ± 17.50	3.553	0.001
Observation Group (n = 136)	Pre-treatment	84.56 ± 20.90	—	—
	Post-treatment	68.76 ± 17.96	6.687	< 0.001
Between-group difference	Pre-treatment	8.28 ± 5.20	1.865	0.065
	Post-treatment	16.32 ± 4.35	3.647	< 0.001

SD, Standard Deviation.

— indicates no statistical test was performed within group at baseline.

### 3.3 PEP-3 domain score improvements

Both groups demonstrated improvements across PEP-3 domains following intervention. In the cognitive domain, the control group’s mean score increased from 35.11 ± 16.40 to 43.26 ± 14.18 (P = 0.048), while the observation group rose from 39.98 ± 15.89 to 51.29 ± 12.47 (P = 0.004). Post-treatment between-group comparison favored the observation cohort (difference 8.03 ± 3.31; P = 0.026). Affective expression improved from 12.69 ± 2.97 to 14.55 ± 3.06 in controls (P = 0.011) and from 13.91 ± 2.36 to 16.23 ± 2.19 in the observation group (P < 0.001). Although the between-group difference was not significant (P = 0.130), trends favored combined intervention. Social interaction scores rose significantly in the control group (11.43 ± 4.06 to 13.51 ± 4.08; P = 0.048) and non-significantly in the observation group (13.49 ± 4.22 to 15.27 ± 3.89; P = 0.102). The post-treatment between-group difference (1.76 ± 1.08) did not reach statistical significance (P = 0.131) (**Table 3**).

**Table 3:** Comparison of PEP-3 domain scores before and after treatment between groups (mean ± SD).

Domain	Group/comparison	Time point	Score (mean ± SD)	t Value	P Value
Cognitive	Control Group	Pre-treatment	35.11 ± 16.40	—	—
		Post-treatment	43.26 ± 14.18	2.012	0.048
	Observation Group	Pre-treatment	39.98 ± 15.89	—	—
		Post-treatment	51.29 ± 12.47	3.014	0.004
	Between-group comparison	Pre-treatment	4.87 ± 4.07	1.433	0.154
		Post-treatment	8.03 ± 3.31	2.284	0.026
Affective Expression	Control Group	Pre-treatment	12.69 ± 2.97	—	—
		Post-treatment	14.55 ± 3.06	2.603	0.011
	Observation Group	Pre-treatment	13.91 ± 2.36	—	—
		Post-treatment	16.23 ± 2.19	4.002	<0.001
	Between-group comparison	Pre-treatment	1.22 ± 1.07	1.864	0.067
		Post-treatment	1.68 ± 1.06	1.537	0.13
Social Interaction	Control Group	Pre-treatment	11.43 ± 4.06	—	—
		Post-treatment	13.51 ± 4.08	2.006	0.048
	Observation Group	Pre-treatment	13.49 ± 4.22	—	—
		Post-treatment	15.27 ± 3.89	1.655	0.102
	Between-group comparison	Pre-treatment	2.06 ± 1.21	1.864	0.067
		Post-treatment	1.76 ± 1.08	1.537	0.131

PEP-3, Psycho-Educational Profile, Third Edition; SD, Standard Deviation.

— indicates no statistical test was performed within group at baseline.

### 3.4 PEP-3 domain score comparisons

Both groups showed significant post-treatment improvements in motor and imitation domains: fine motor (Control: P = 0.014; Observation: P = 0.041), gross motor (Control: P = 0.003; Observation: P = 0.019), and imitation (Control: P = 0.009; Observation: P = 0.001). Reductions in problem behaviors favored the observation group (P = 0.036). Changes in language expression, language understanding, affective expression, social interaction, and behavioral traits did not reach significance (P > 0.05). Baseline scores were comparable across all domains (**Table 4**). Because multiple comparisons were conducted across PEP-3 subdomains, the Benjamini–Hochberg false discovery rate (FDR) procedure was applied. After adjustment, improvements in the cognitive domain and reductions in problem behaviors remained statistically significant, whereas several borderline results did not survive correction. Detailed uncorrected P-values and FDR-adjusted q-values are provided in **Supplementary Table 3**.

**Table 4:** Mann–Whitney U test for PEP-3 domain scores between groups before and after treatment.

Domain	Group	Time point	M (QL, QU)	Z Value	P Value
Language Expression	Control	Pre	18.00 (10.25, 22.00)	—	—
	Control	Post	21.00 (13.00, 25.75)	1.169	0.242
	Observation	Pre	21.50 (4.00, 28.75)	—	—
	Observation	Post	24.50 (10.00, 32.75)	1.539	0.124
	Between-groups	Pre	—	0.583	0.559
	Between-groups	Post	—	0.754	0.453
Language Understanding	Control	Pre	23.00 (16.25, 27.75)	1.753	0.081
	Control	Post	27.50 (22.50, 31.75)	—	—
	Observation	Pre	26.00 (13.25, 30.00)	2.288	0.022
	Observation	Post	31.50 (20.50, 36.00)	—	—
	Between-groups	Pre	—	0.754	0.452
	Between-groups	Post	—	1.323	0.185
Fine Motor	Control	Pre	28.50 (22.25, 33.00)	—	—
	Control	Post	33.00 (31.00, 37.00)	2.455	0.014
	Observation	Pre	30.00 (22.00, 35.75)	—	—
	Observation	Post	35.50 (28.50, 38.75)	2.046	0.041
	Between-groups	Pre	—	0.651	0.515
	Between-groups	Post	—	0.549	0.583
Gross Motor	Control	Pre	23.00 (22.00, 26.00)	2.996	0.003
	Control	Post	28.00 (24.25, 28.00)	—	—
	Observation	Pre	25.00 (22.00, 29.00)	2.35	0.019
	Observation	Post	29.00 (26.50, 30.00)	—	—
	Between-groups	Pre	—	1.084	0.278
	Between-groups	Post	—	1.954	0.051
Imitation	Control	Pre	14.50 (8.25, 17.00)	2.627	0.009
	Control	Post	18.00 (14.00, 19.00)	—	—
	Observation	Pre	16.00 (12.00, 18.00)	3.242	0.001
	Observation	Post	19.00 (16.00, 20.00)	—	—
	Between-groups	Pre	—	1.174	0.243
	Between-groups	Post	—	1.288	0.198
Behavioral Traits – Nonverbal	Control	Pre	20.00 (17.25, 24.00)	1.558	0.119
	Control	Post	23.00 (20.00, 26.00)	—	—
	Observation	Pre	23.00 (18.00, 27.00)	1.838	0.066
	Observation	Post	25.00 (21.25, 27.75)	—	—
	Between-groups	Pre	—	1.141	0.254
	Between-groups	Post	—	1.624	0.104
Behavioral Traits – Verbal	Control	Pre	7.50 (3.00, 12.75)	1.869	0.067
	Control	Post	11.50 (8.00, 14.75)	—	—
	Observation	Pre	11.00 (3.00, 15.00)	1.663	0.096
	Observation	Post	15.00 (6.00, 18.00)	—	—
	Between-groups	Pre	—	1.163	0.248
	Between-groups	Post	—	1.148	0.251
Problem Behaviors	Control	Pre	9.00 (6.25, 10.75)	1.31	0.19
	Control	Post	10.00 (7.50, 11.75)	—	—
	Observation	Pre	9.00 (6.25, 10.00)	2.651	0.008
	Observation	Post	12.00 (10.00, 15.00)	—	—
	Between-groups	Pre	—	0.476	0.634
	Between-groups	Post	—	2.099	0.036
Personal Autonomy	Control	Pre	16.00 (13.00, 20.75)	1.342	0.185
	Control	Post	19.00 (15.00, 21.00)	—	—
	Observation	Pre	17.00 (14.00, 20.00)	1.266	0.211
	Observation	Post	18.50 (15.00, 22.00)	—	—
	Between-groups	Pre	–	0.533	0.594
	Between-groups	Post	–	0.267	0.793
Adaptive Behavior	Control	Pre	18.00(14.25)	0.912	0.362
	Control	Post	19.00(15.50)		
	Observation	Pre	17.00(15.25)	1.033	0.306
	Observation	Post	18.00(15.25)		
	Between-groups	Pre	—	0.267	0.792
	Between-groups	Post	—	-0.379	0.705

— indicates no statistical test was performed within group at baseline.

M, median; QL, lower quartile; QU, upper quartile.

### 3.5 ATEC and PEP-3 score difference comparison

The observation group showed significantly greater median improvement in total ATEC scores (12.00 *vs*. 3.00; P < 0.001) and in PEP-3 cognition (9.00 *vs*. 5.00; P = 0.004) and problem behavior (2.00 *vs*. 0.00; P < 0.001) compared with controls. Changes in all other PEP-3domains did not differ significantly between groups (P > 0.05) (**Table 5**). Rank-biserial effect sizes supported these findings (r = 0.223 for ATEC, r = 0.180 for PEP-3 cognition, and r = 0.259 for problem behavior), consistent with greater improvement in the ESDM+TEACCH group (**Supplementary Table 4**).

**Table 5:** Comparison of ATEC and PEP-3 score differences between control and observation groups pre- and post-treatment.

Domain	Control group/M (QL, QU)	Observation group/M (QL, QU)	Z Value	P Value
ATEC Total Score Difference	3.00 (2.00, 6.00)	12.00 (6.00, 21.50)	3.620	<0.001*
PEP-3 Domains				
- Cognition	5.00 (2.25, 11.75)	9.00 (7.25, 13.75)	2.920	0.004*
- Language Expression	3.50 (2.00, 4.00)	4.00 (2.25, 5.75)	1.437	0.151
- Language Comprehension	3.50 (1.25, 5.75)	5.50 (3.25, 7.00)	1.718	0.085
- Fine Motor Skills	4.00 (1.25, 8.00)	3.50 (2.00, 5.75)	0.712	0.480
- Gross Motor Skills	3.00 (1.00, 5.00)	2.00 (0.00, 5.75)	0.592	0.554
- Imitation	3.00 (2.00, 4.00)	2.00 (2.00, 4.00)	0.473	0.636
- Affective Expression	2.00 (1.25, 2.00)	2.00 (1.25, 3.00)	0.997	0.319
- Social Interaction	2.00 (1.00, 3.00)	2.00 (1.00, 2.00)	0.804	0.421
- Behavioral Traits (Nonverbal)	1.00 (1.00, 3.00)	2.00 (1.00, 4.00)	0.580	0.562
- Behavioral Traits (Verbal)	2.00 (0.25, 2.75)	2.50 (1.00, 3.75)	0.931	0.352
- Problem Behavior	0.00 (0.00, 2.00)	2.00 (2.00, 4.00)	4.209	<0.001*
- Personal Autonomy	1.00 (0.00, 3.75)	1.00 (1.00, 2.00)	0.319	0.750
- Adaptive Behavior	0.00 (0.00, 2.00)	1.00 (0.00, 2.00)	0.114	0.913

TEC, Autism Treatment Evaluation Checklist; PEP-3, Psycho-Educational Profile, Third Edition; M, median; QL, lower quartile; QU, upper quartile.

### 3.6 Power calculation

To evaluate the adequacy of the sample size for detecting meaningful differences in primary and secondary outcomes, a *post-hoc* power analysis was performed. Under a two-sided α = 0.05 and assuming 80% power, the minimum detectable effect (MDE) for this study was calculated as Cohen’s d ≈ 0.345, which corresponds to approximately 6.12 points for ATEC and 4.60 points for PEP-3 cognition. The observed between-group differences at follow-up were ATEC ≈ 16.15 points and PEP-3 cognition ≈ 8.03 points, both of which exceed the MDE thresholds, indicating that the sample size provided at least 80% power to detect significant differences for the primary endpoints.

## 4 Discussion

The present study evaluated the effectiveness of combining ESDM with the TEACCH program in young children with ASD. ATEC total scores decreased significantly in both the control and observation groups, with a larger reduction in the latter. On the PEP-3, significant within-group gains were observed for cognition (control: P = 0.048; observation: P = 0.004), fine and gross motor skills, and imitation (all P ≤ 0.041). In contrast, changes in language expression/understanding and social interaction in the observation group did not reach statistical significance (e.g., social interaction P = 0.102). Post-treatment between-group comparisons favored the observation group for cognition (P = 0.026), whereas differences in social interaction were not significant (P = 0.131). Because multiple subdomains were assessed, we applied the Benjamini–Hochberg FDR procedure; after correction, improvements in cognition and reductions in problem behaviors remained significant, while several borderline findings did not survive adjustment.

Regarding developmental domains assessed using the PEP-3, both groups exhibited significant within-group improvements. However, the observation group demonstrated more pronounced gains in cognitive development. Cognition scores increased from approximately 40 to 51 points in the observation group, compared to a more modest increase from 35 to 43 points in the control group. This between-group difference was statistically significant and clinically relevant, given the role of early cognitive ability in predicting adaptive functioning and academic success in ASD. In addition, the combined-treatment group exhibited significantly greater improvements in maladaptive behaviors than the control group. While both groups showed improvements, the observation group achieved significantly larger median improvement. TEACCH is known to manage problem behaviors by promoting environmental structure and visual organization. The incorporation of ESDM techniques, including positive reinforcement and teaching alternative behaviors, likely reinforced these effects by improving communication and reducing frustration. This integrated approach may have created a more supportive and responsive learning environment (4, 18). Notably, the combined intervention also yielded greater reductions in ATEC total scores compared to ESDM alone, further supporting its superior efficacy in alleviating core ASD symptoms. Other developmental domains, such as language expression and comprehension, social interaction, and affective expression, improved similarly in both groups without statistically significant between-group differences. These results suggest that TEACCH alone may be effective in promoting communication and social-emotional development in early ASD intervention. The absence of additional benefit from ESDM in these domains may be due to limited study duration, ceiling effects, or insufficient sensitivity of the assessment tools. Nonetheless, the combined approach showed a broader pattern of improvement, particularly in cognitive functioning and symptom reduction, underscoring the potential advantage of multifaceted interventions (19, 20).

Comparison with previous research provides further support for the current findings. ESDM has been widely studied and recognized for its effectiveness in enhancing cognitive and language outcomes in young children with ASD. Studies such as those by Dawson et al. and Zhou et al. have demonstrated substantial IQ gains and improvements in language development following intensive ESDM intervention (18, 21). These findings parallel the significant cognitive improvements observed in the observation group in our study. Additionally, prior research has shown that ESDM can reduce autism symptom severity, aligning with the greater improvements in ATEC scores noted in the combined-treatment group (22). Conversely, TEACCH has a more varied evidence base. Meta-analyses and empirical studies have suggested that while TEACCH may offer moderate benefits in reducing problem behaviors and enhancing social engagement, its impact on cognitive and language development tends to be more limited. The findings from our study support this interpretation, as the control group showed measurable but smaller gains in cognition and no significant advantage in language or social interaction. This suggests that the structured teaching principles of TEACCH, particularly in managing maladaptive behaviors, may complement the developmental focus of ESDM, resulting in a more comprehensive intervention strategy (23).

Our results extend existing literature by demonstrating that integrating two evidence-based programs with distinct emphases can result in additive benefits. While previous studies, such as the TADPOLE trial, found comparable outcomes among different behavioral interventions, our findings suggest that combining programs with complementary strengths, namely ESDM’s developmental-behavioral framework together with TEACCH’s structured teaching, can yield superior outcomes, particularly in cognitive functioning and behavioral regulation. From a clinical perspective, the study supports the utility of integrated intervention models in early ASD treatment. Practitioners should consider blending strategies from ESDM and TEACCH to address the diverse needs of autistic children. TEACCH provides a predictable, low-stress learning environment, while ESDM promotes engagement, communication, and learning through play-based interaction (24, 25). Together, these approaches can create a responsive and enriched setting conducive to development across multiple domains. Training educators and therapists in both methods may enhance program effectiveness and allow for individualized application based on each child’s profile. Moreover, the observed improvements suggest that early implementation of a combined intervention may enhance school readiness and long-term developmental outcomes. Parental involvement in applying consistent strategies at home can further reinforce gains made in therapy settings, emphasizing the importance of family-centered care (26, 27). Given the robust improvements in cognition and symptom severity, integrating TEACCH into existing ESDM-based programs may represent a feasible and effective approach in clinical practice.

Several limitations should be acknowledged. First, the retrospective, non-randomized design with sequential group assignment across different time periods introduces a risk of temporal confounding. Although baseline demographic and clinical characteristics were comparable, unmeasured changes in clinical practices, therapist experience, or environmental context (e.g., post-COVID factors) may have influenced outcomes. Adjusted analyses using ANCOVA and sensitivity analyses based on propensity score weighting were conducted to mitigate these biases, and results remained consistent. Nevertheless, residual confounding cannot be fully excluded. Second, intervention fidelity was not formally quantified. Although therapists were trained and supervised in both ESDM and TEACCH protocols, and session records were regularly reviewed, the absence of prospective fidelity scoring (e.g., video review or inter-rater reliability) limits the ability to assess consistency of implementation across participants. Third, several baseline covariates such as socioeconomic status, comorbidities, and family history were retrospectively extracted from clinical records and may have been incompletely documented, which reduced our capacity to fully adjust for potential confounders. Fourth, outcome evaluation partly relied on parent-reported measures (e.g., ATEC), which are susceptible to expectancy and reporting bias, particularly in the absence of blinding. While standardized tools like the PEP-3 were also used, caregiver perceptions may have influenced observed differences. Therapists and caregivers were not blinded to intervention type, which may have introduced additional bias. Fifth, the relatively short follow-up period precludes conclusions about the long-term durability of treatment effects. Additionally, not all developmental domains showed significant between-group differences, which may reflect limited intervention duration, ceiling effects of assessment tools, or insufficient statistical power. Finally, concurrent implementation of both ESDM and TEACCH requires substantial time and institutional resources, potentially limiting feasibility in low-resource settings. Future research should prioritize randomized controlled trials with prospective fidelity monitoring, blinded assessments, and longer-term follow-up. Stratified analyses may also help identify subgroups that derive the greatest benefit from integrated interventions.

## 5 Conclusions

In conclusion, the combined application of the ESDM and the TEACCH program demonstrated superior efficacy in improving cognitive development and reducing autism symptom severity in young children with ASD compared to ESDM alone. These findings support the clinical value of integrated intervention models in enhancing early developmental outcomes for children on the autism spectrum.

## Data Availability

The raw data supporting the conclusions of this article will be made available by the authors, without undue reservation.
