# Supplementary material for: Efficacy of the early start Denver model combined with the TEACCH program in children with autism spectrum disorder
Source: Front Psychiatry. 2025 Nov 18;16:1669476. doi: 10.3389/fpsyt.2025.1669476 (PMC12669128; doi:10.3389/fpsyt.2025.1669476)
Supplement: Supplementary file 1 [file Table1.docx]

Supplementary Table 1. Adjusted and Sensitivity Analyses for Primary Outcomes.

| Outcome | Analysis Type | Mean Difference (95% CI) | P-value | Significance |
| --- | --- | --- | --- | --- |
| ATEC score reduction | Unadjusted | –16.3 (–20.6 to –11.9) | <0.001 | Significant |
|  | ANCOVA (adjusted for baseline, age, sex) | –12.4 (–16.8 to –7.9) | <0.001 | Significant |
|  | IPTW (propensity score weighted) | –11.9 (–16.2 to –7.6) | <0.001 | Significant |
| PEP-3 cognition | Unadjusted | +8.0 (3.3 to 12.7) | 0.026 | Significant |
|  | ANCOVA (adjusted) | +7.1 (3.2 to 11.0) | 0.001 | Significant |
|  | IPTW (weighted) | +6.8 (2.7 to 10.9) | 0.002 | Significant |
| Problem behaviors | Unadjusted | –2.0 (–3.6 to –0.4) | 0.036 | Significant |
|  | ANCOVA (adjusted) | –2.3 (–3.9 to –0.8) | 0.003 | Significant |
|  | IPTW (weighted) | –2.1 (–3.7 to –0.6) | 0.005 | Significant |

ATEC = Autism Treatment Evaluation Checklist; PEP-3 = Psycho-Educational Profile, Third Edition; ANCOVA = analysis of covariance; IPTW = inverse probability of treatment weighting.

Supplementary Table 2. Between-group effect size (ESDM+TEACCH vs. ESDM) at post-treatment

| Outcome | Comparison | n (control) | n (observation) | Test statistic | Cohen’s d | Hedges’ g (bias-corrected) | 95% CI for g |
| --- | --- | --- | --- | --- | --- | --- | --- |
| ATEC total (post-treatment) | Observation – Control | 128 | 136 | t = 3.647 | 0.45 | 0.45 | 0.20 to 0.69 |

Supplementary Table 3. PEP-3 Subdomain Results Before and After FDR Correction.

| Domain | Uncorrected P-value | FDR-adjusted q-value | Significance After FDR |
| --- | --- | --- | --- |
| Cognition | 0.026 | 0.041 | Significant |
| Problem behaviors | <0.001 | <0.001 | Significant |
| Affective expression | 0.048 | 0.072 | Not significant |
| Fine motor skills | 0.041 | 0.065 | Not significant |
| Gross motor skills | 0.051 | 0.078 | Not significant |
| Social interaction | 0.131 | 0.169 | Not significant |
| Language expression | 0.151 | 0.192 | Not significant |
| Language comprehension | 0.085 | 0.124 | Not significant |
| Behavioral traits (NV) | 0.562 | 0.614 | Not significant |
| Behavioral traits (V) | 0.352 | 0.398 | Not significant |
| Personal autonomy | 0.750 | 0.782 | Not significant |
| Adaptive behavior | 0.913 | 0.941 | Not significant |

FDR = false discovery rate; NV = nonverbal; V = verbal.

Supplementary Table 4. Effect sizes for between-group differences in change scores (Δ = post − baseline)

| Domain (Δ) | Test | Z | Rank-biserial r | Approx. Cohen’s d |
| --- | --- | --- | --- | --- |
| ATEC total (Δ) | Mann–Whitney U | 3.62 | 0.223 | 0.46 |
| PEP-3 Cognition (Δ) | Mann–Whitney U | 2.92 | 0.18 | 0.37 |
| PEP-3 Problem Behavior (Δ) | Mann–Whitney U | 4.209 | 0.259 | 0.54 |
